# L-Dopa Medication in Parkinson's Disease Restores Activity in the Motor Cortico-Striatal Loop but Does Not Modify the Cognitive Network

**DOI:** 10.1371/journal.pone.0006154

**Published:** 2009-07-07

**Authors:** Thomas Jubault, Laura Monetta, Antonio P. Strafella, Anne-Louise Lafontaine, Oury Monchi

**Affiliations:** 1 Unité de Neuroimagerie Fonctionnelle, Institut Universitaire de Gériatrie de Montréal, Montreal, Québec, Canada; 2 Department of Radiology, University of Montreal, Montreal, Québec, Canada; 3 Toronto Western Hospital/Research Institute & CAMH-PET Imaging Centre, University of Toronto, Toronto, Ontario, Canada; 4 Movement Disorders Unit, McGill University Health Centre, Toronto, Ontario, Canada; The University of Western Ontario, Canada

## Abstract

**Background:**

The goal of this study was to evaluate the effects of L-Dopa medication in Parkinson's disease (PD) on brain activation during the performance of a set-shifting task. Using fMRI, we have previously studied the patterns of activity observed in patients with PD after overnight removal of dopaminergic medication compared with control participants during the performance of different stages of the Wisconsin Card Sorting Task (WCST). The results revealed decreased cortical activity in the PD group compared to controls in the conditions that significantly required striatum, while increased cortical activity was observed when striatum was not involved. However, the effect of dopaminergic medication in PD patients on those patterns of activity has not yet been studied.

**Methodology/Principal Findings:**

Here, eleven PD patients at early stage of the disease taking L-Dopa medication were recruited and underwent two fMRI sessions while performing the WCST: one session while taking their normal dose of medication and the other following overnight dopaminergic medication withdrawal. We found that L-dopa medication helped restoring a normal pattern of activity when matching and not planning was required, by increasing cortical activity in the premotor cortex. This effect was even stronger in the motor loop, i.e. when the putamen was required for controls, when matching following negative feedback. However, the medication did not change the pattern of activity in conditions relying primarily on a cognitive loop, i.e. when the caudate nucleus was required.

**Conclusions/Significance:**

These studies provide explanation at the neural level regarding the relatively poor effects of L-Dopa on the cognitive deficits observed in PD.

## Introduction

Patients suffering from PD exhibit a specific array of motor symptoms, but a wide range of non-motor deficits can also appear in the course of the disease; including cognitive changes which can lead to a full-blown dementia [1]. These cognitive impairments resemble those observed in patients with frontal lesions [2], as PD patients seem to be particularly impaired at tasks relying on executive functions, such as the Wisconsin Card Sorting Task (WCST) [3]. The origin of these impairments is still controversial. Some authors have hypothesized them to originate from a disruption of striatal outflow [4], itself caused by dopaminal depletion and resulting in frontal dysfunction through the general unbalance of the cortico-striatal loops [5]. Others have proposed that they may result from an overactive dopaminergic tone in the prefrontal cortex via the meso-cortical pathway [6], [7]. We have previously proposed that both may actually occur depending on the striatal requirement for the task [8], [9].

In a previous study with healthy subjects, we investigated the functional contributions of distinct cortico-striatal circuits to various stages of the WCST, using event related 1.5T fMRI [10]. The “cognitive” fronto-striatal loop [5], including the caudate nucleus and the prefrontal cortex (PFC) was activated when *planning* a set shift, while the “motor” fronto-striatal loop, including the putamen and the premotor cortex (PMC, areas 6, 8) was found when *executing* a set-shift [10]. These results were recently reproduced [11] using the 3T MRI scanner we used in the present study. We used the same fMRI protocol in PD patients OFF dopaminergic medication and matched controls to study the effect of the disease on the aforementioned fronto-striatal networks [8]. The premotor cortex of PD patients exhibited decreased activity only when the putamen was required for the task in controls, and in the ventrolateral prefrontal cortex (VLPFC) when the caudate nucleus is required. On the other hand, increased activation was found in various prefrontal regions in the PD patients vs. controls for conditions not requiring the striatum. This disruption of the motor and cognitive loops might explain at a functional level the cognitive impairments that progressively appear in PD. However, the effect of L-Dopa medication on these two loops remains poorly understood.

The most common treatment of PD is based on the administration of L-Dopa. In the majority of PD patients, the improvement of the motor symptoms is often spectacular. Little is known, however, about the effect of L-Dopa on cognitive deficits and studies have reported them to be eitherbeneficial [12] or deleterious [13], [14].

The goal of the present study was to assess whether, and to what extent L-Dopa medication restores normal patterns of activation in the cognitive and motor cortico-striatal loops that have been identified respectively for the planning and the execution of set-shifting processes in the WCST. Based on the striatal-dependent patterns of activity we observed in PD OFF medication vs. controls [8], we predicted L-Dopa to act significantly more on cortical regions that co-activate with the striatum during the task (such as the PMC when executing a set-shift) than on cortical regions that do not. Furthermore we expected the effect to be more pronounced for the periods of the task that relies on motor networks (i.e. the matching periods) than cognitive ones (i.e. the feedback periods).

## Materials and Methods

### Participants

11 patients diagnosed with Parkinson's disease (mean age, 63.9 years; range: 55–78, 4 females and 7 males, 10 right handed and 2 ambidextrous) participated in the study (see table 1). All participants met the core assessment program for surgical interventional therapy criteria for the diagnosis of idiopathic PD [15], [16], namely two of the three cardinal signs of PD (bradykinesia, tremor, rigidity), response to L-dopa, and lack of evidence of other medical conditions associated with Parkinsonism. Motor disability of individuals within the PD group was in the mild to moderate severity range according to the Hoehn and Yahr staging criteria [17]. All patients were medicated with levodopa–carbidopa (n = 11), and were taking on average 523 mg of L-Dopa per day. Some patients were also taking other antiparkinsonian medications as follows: dopamine agonists/Pramipexole (n = 4), MAO-B inhibitor/Selegiline (n = 2), COMT inhibitor (n = 4). All individuals were screened for dementia prior to the experiment using the Montreal Cognitive Assessment [18] (mean average ON 25.4, OFF 26.2). The presence and severity of depression in all PD participants was estimated using the Beck Depression Inventory (BDI) (mean average ON 7.8, OFF 7.9).

**Table 1:** subjects information.

Subj.	gender	age	lat.	Years since diagnosis	L-Dopa daily intake (mg)	Other anti-parkinsonian med.			Time since last med. intake (h)	UPDRS	
						comT inhibitor	MAO-B inhibitor	DA agonist		On	Off
1	F	67	R	10	700				14	15	23
2	F	55	R	13	700	•			12	19	34
3	F	59	R	5	300				13	16	25.5
4	F	56	R	5	150		•	•	15	14.5	31.5
5	M	73	R	2	400	•			17	22.5	35
6	M	57	A	3	1200			•	12	25	29
7	M	66	R	2	550				12	13	24.5
8	M	67	R	8	450		•	•	12	23.5	34
9	M	78	R	10	700				12	25.5	33.5
10	M	57	A	9	300	•			17	12	23
11	M	68	R	6	300	•		•	15	25	32
**Average**		**63.9**		**6.6**	**522.7**				**13.7**	**19.2**	**29.5**

Abbreviations: Subj.: subject, F: female, M: male, lat.: laterality, R: right-handed, A: ambidextrous, med.: medication, DA: dopamine.

#### Ethics statements

All participants gave informed consent to the protocol, which was reviewed and approved by the Joint Ethics Committee of the Regroupement Neuroimagerie Quebec, which follows the guidelines of the Tri-Council Policy Statement of Canada, the civil code of Quebec, the Declaration of Helsinki and the code of Nuremberg.

#### Cognitive task

The same version of the WCST we have used previously [8], [10] was administered using a customized software, and a full description of the task can be found in those reports. Briefly, throughout the task, four fixed reference cards were present in a row in the upper part of the screen, displaying a red rectangle, two green stars, three yellow crosses and four blue circles. Participants used a 2 buttons response-box with their right hand (index and middle finger). The index button moved a cursor along the four reference cards, and the middle finger confirmed the choice. On each test trial, a new card was presented. Subjects were required to match the test card to one of the reference card according to the color, the shape or the number of items shared by the test and reference cards. The rule for classification was not explicit and had to be found using feedback (positive or negative) that followed each trial. On each experimental trial, participants had to find the proper classification rule and apply it as long as a positive feedback followed the selection. A change in the screen brightness reflected a correct (bright screen) or incorrect (dark screen) answer. On each control trial, the test card was identical to one of the four reference cards, and therefore participants only had to select the twin reference card.

We defined six experimental time periods: three feedback periods: negative, positive, or control feedback and three corresponding matching periods i.e. matching after negative, positive, or control feedback. Each feedback period lasts 2.30 s, and the length of each matching period depends on the subject's response time.

Activity in the appropriate period of the control trials was subtracted from that of the different experimental event periods for the color, shape, and number of items trials to generate the following four contrasts for statistical analysis: (1) receiving negative feedback minus control feedback; (2) matching after negative feedback minus control matching; (3) receiving positive feedback minus control feedback and (4) matching after positive feedback minus control matching.

### Procedure

All participants came for two scanning sessions within two weeks, once OFF their prescribed antiparkinsonian medications for at least 12 hours (“OFF” state) and the other time ON their usual medications (“ON” state). ON and OFF sessions order was counter-balanced across participants. Prior to each session, participants were evaluated for their motor symptoms with the UPDRS III (ON: 19.2 (sd 5.3), OFF:29.5 (sd 4.7), p<0.001).

All participants were trained on the task prior to each the scanning session for at least 30 minutes and until no additional improvement could be observed. Each scanning session consisted in five functional runs. Within each run, blocks of each of the four trial types (color, shape, number and control) were presented in random order, so that no trial type could be repeated before all four trial types had occurred. In the experimental WCST trials blocks, six correct matching responses had to be completed in a row before a rule change occurred. The control blocks contained eight trials each.

### fMRI scanning

#### Data acquisition

Subjects were scanned using the 3T Siemens Trio MRI scanner at the Functional Neuroimaging Unit, at the Research Center of the Montreal Geriatric's Institute. Each scanning session began with a T1-weighted volume acquisition for anatomical localization (voxel size, 1 mm^3^). This was followed by acquisitions of echoplanar T2*-weighted images with blood oxygenation level-dependent contrast (echo time, 30 msec; flip angle, 90°). 155 volumes containing 36 slices (matrix size, 64×64 pixels, voxel size 3.7×3.7×3.7 mm3) were acquired continuously every 2.5 sec within each run. Stimulus presentation and scanning were synchronized at the beginning of each run.

#### Data analysis

Data analysis were performed with the fmristat software, developed by Worsley et al. [19] (http://www.math.mcgill.ca/keith/fmristat/) similarly to our previous studies [20] and was based on was based on a linear model with correlated errors. The design matrix of the linear model was first convolved with a difference of two gamma hemodynamic response functions timed to coincide with the acquisition of each slice. The significance of peaks is reported using the minimum p value of the single peak analysis and cluster analysis. All peaks that reached p<0.05 corrected are reported. Predicted peaks that reached p<0.0001 uncorrected are also reported, and are shown with a * in the tables. A region was predicted if it was significant in our study using the same fMRI protocol on PD and healthy subjects [8].

## Results

### Behavioural performance

Patients in their ON state completed an average of 25.7 (OFF: 29.7) experimental WCST trial blocks and 9.2 (OFF: 9.9) control blocks during the five runs. They made on average 1.45 (OFF: 0.86) perseverative errors (i.e. errors attributable to the fact that the subject incorrectly used the same classification rule after negative feedback) and 0.78 (OFF: 1.00) non-perseverative errors (loss of the rule) per WCST trial bloc. They made an average of 1.85 (OFF: 1.82) incorrect classifications per experimental WCST trial block after a change in the rule – but the later were not considered as errors because subjects could not know the new classification rule on the first attempt after a set shift. None of these behavioral differences reached significance between the ON and OFF states.

### fMRI results

As predicted, a significant effect of L-Dopa was only found in the condition when a motor cortico-striatal loop was required for the task at hand [8], [10]. Indeed, significantly increased activity in the ON vs. OFF state was observed during matching following feedback vs. control matching, while no other significant activation was observed in any of the other three subtractions in the ON vs. OFF state. Below, both the results for the intra-group analysis (ON and OFF separately), and the inter-group analysis (ON vs. OFF, and OFF vs. ON) are reported for each of the four contrasts of interest. It should be noted that the patterns of activation observed in the present study during the OFF state were similar to those observed in our previous study [8] using the same protocol in another group of PD patients OFF with a 1.5T scanner. The dorsolateral prefrontal cortex (DLPFC), the posterior PFC and the anterior cingulate gyrus were observed during the reception of negative feedback, and the DLPFC when matching following negative feedback.

### 1) Receiving negative feedback vs. control feedback

**Figure 1:**
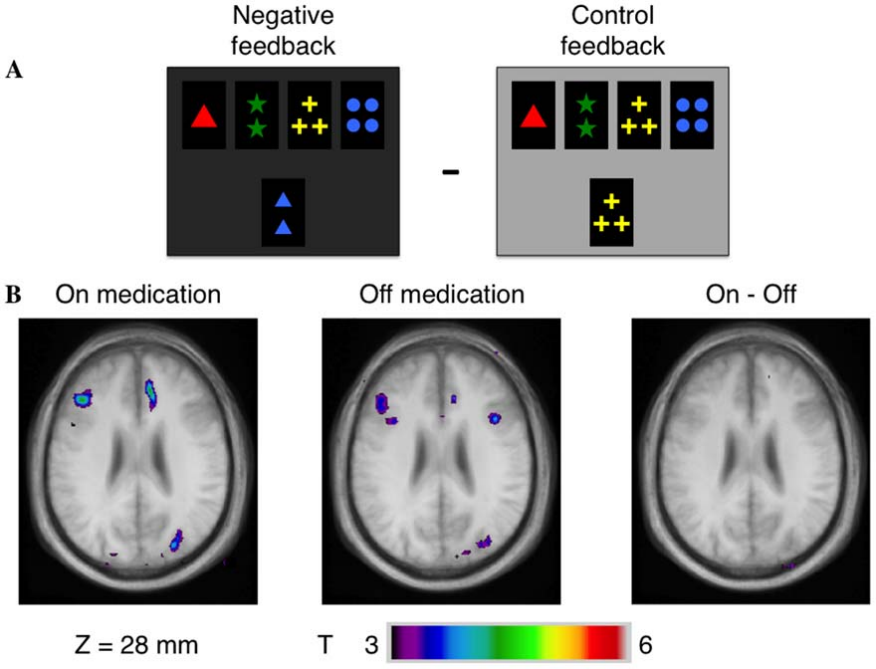
Patterns of activation in the left VLPCF and right cingulated cortex when receiving negative feedback compared to control feedback. A – appearance of the monitor when receiving negative feedback and control feedback. B – axial section (z = 28 mm) in the ON and OFF groups and in the intergroup analysis showing greater activations in the ON – OFF comparison.

**ON state:** (Table 2, Figure 1), we found significant activity increases bilaterally in the prestriate cortex (area 19). There were also increases of activity in the left hemisphere in the DLPFC (area 9,46), in the PMC (area 6) and in the right hemisphere, in the anterior cingulate cortex (area 32). **OFF state:** only subtle differences were notable, as there was no increase of activity in the anterior cingulate cortex, but additional activity in the left posterior parietal cortex (PPC, area 7). **Intergroup comparison:** we found no significant difference of activity.

**Table 2:** Receiving negative feedback minus control feedback.

Anatomical area		ON medication			OFF medication			
		x, y, z	t-stat	Cluster	x, y, z	t-stat	Cluster	
Anterior CC	(32) R	10, 34, 28	4.49	1016	10, 30, 30	4.09*	168	
Posterior PFC	(6,44) R				42, 14, 28	3.94*	336	
PMC	(6) L	−44, 6, 40	3.69*	256				
DLPFC	(46) L	−42, 28, 26	4.50	2496	−40, 34, 14	4.01	3016	
	(9) L	−42, 22, 38	4.09	sc				
PPC	(7) L				−20, −78, 52	3.91	592	
pcu	(7)				2, −80, 42	3.99*	432	
Prestriate cortex	(17) L	−6, −76, 8	4.84	>10000	−6, −88, 4	4.71	>10000	
	(17) R	10, −66, 10	5.21	sc	18, −80, 6	4.20	sc	
	(18) L	−16, −88, 14	3.70	sc	−6, −66, 2	5.79	sc	
	(18) R	28, −74, −8	6.45	sc	26, −72, −6	5.16	sc	
	(19) L	−16, −88, 24	3.65	sc	−38, −84, 4	4.82	1808	
	(19) R	30, −78, 24	4.46	sc	36, −84, 4	4.97	2976	
		ON greater than OFF			OFF greater than ON			
		-	-	-	-	-	-	

Abbreviations: L, Left; R, right; DL, dorsolateral; VL, ventrolateral; CC, cingulate cortex; PMC, premotor cortex; PPC, posterior parietal cortex; TC, temporal cortex; pSMA, presupplementay motor area. When no asterisk is indicated, p<0.05 corrected; *p<0.001 uncorrected. Cluster sizes are reported in mm3. sc indicates that the peak is part of the same cluster as the peak listed immediately above in the table. The numbers in parentheses refer to architectonic areas.

### 2) Matching after negative feedback vs. control matching

**Figure 2:**
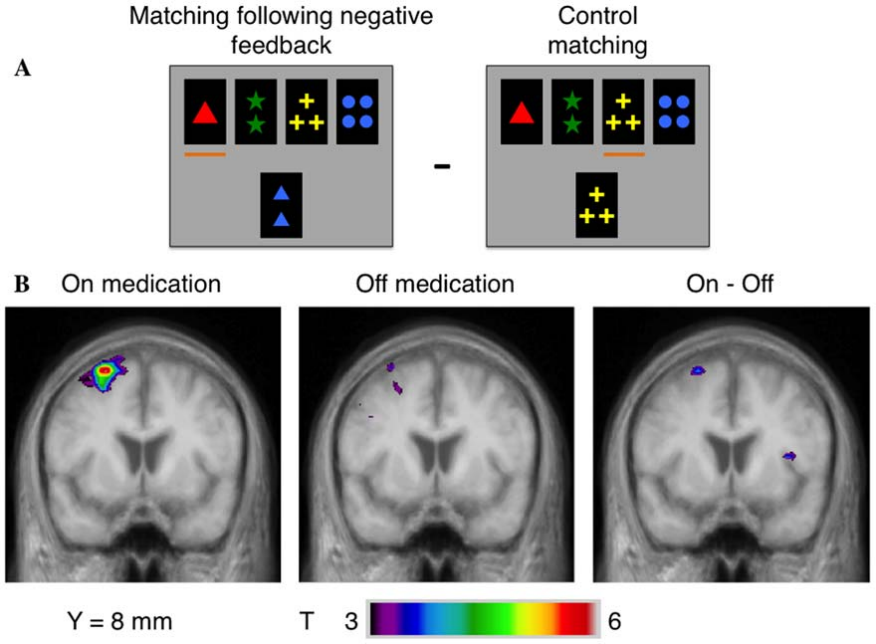
Patterns of activation in the left PMC when matching after negative feedback compared to matching in the control condition. A – appearance of the monitor when matching after negative feedback and matching in the control condition. B – coronal section (y = 8 mm) in the ON and OFF groups and in the intergroup analysis showing greater activations in the ON – OFF comparison.

**ON state:** (Table 3, Figure 2) we found significant activity increases in the left hemisphere in the PMC and supplementary motor area (area 6), in the inferior parietal cortex (area 40), the prestriate cortex (area 19) and temporal cortex (area 39). There were also bilateral increases in the superior parietal lobule and the precuneus (area 7). **OFF state:** bilateral increase of activity was more extended than in the ON state, in the bilateral prestriate cortex (areas 17,18,19) and in the PPC (area 7). By contrast, we found also significant increase of activity in the left DLPFC (area 9,46) but not in the left PMC (area 6) and temporal cortex (area 39). **Intergroup comparison:** the left PMC (area 6) was significantly more activated in the ON than in the OFF state. Conversely, prestriate cortex (area 18,19) was significantly more activated in the OFF than in the ON state.

**Table 3:** Matching after negative feedback minus control matching.

Anatomical area		ON medication			OFF medication		
		*x, y, z*	*t*-stat	Cluster	*x, y, z*	*t*-stat	Cluster
PMC	(6) L	−24, 8, 66	5.96	4464			
pSMA	(6)	0, 16, 56	4.39	1568			
DLPFC	(46) L				−50, 30, 26	4,40	3416
	(9) L				−40, 26, 34	4,32	sc
PPC	(7) L	−14, −72, 48	4.24*	288	−32, −72, 52	5,32	>10000
	(7) R	22, −70, 58	4.32	528	30, −74, 54	4,02	2560
	(40) L	−38, −56, 46	5.66	>10000			
pcu	(7) L	−2, −68, 58	4.74	8240	−2, −72, 66	4,02	2560
	(7) R	8, −70, 58	4.37	sc	6, −74, 56	3,90	sc
TC	(39) L	−54, −66, −14	4.59	5128			
Prestriate cortex	(19) L	−26, −68, 40	4.77	8240	−28, −76, 36	5,33	>10000
	(19) R				30, −84, 26	4,93	1568
	(18) L				−24, −94, −14	4,18	>10000
	(18) R				28, −82, −16	5,50	sc
		ON greater than OFF			OFF greater than ON		
PMC	(6) L	−22, 6, 66	3.85	224			
Prestriate cortex	(18) R				26, −90, −16	4,12	1032
	(19) L				−44, −86, −10	3,84*	280

Abbreviations, same as Table 2.

### 3) Receiving positive feedback vs. control feedback

**Figure 3:**
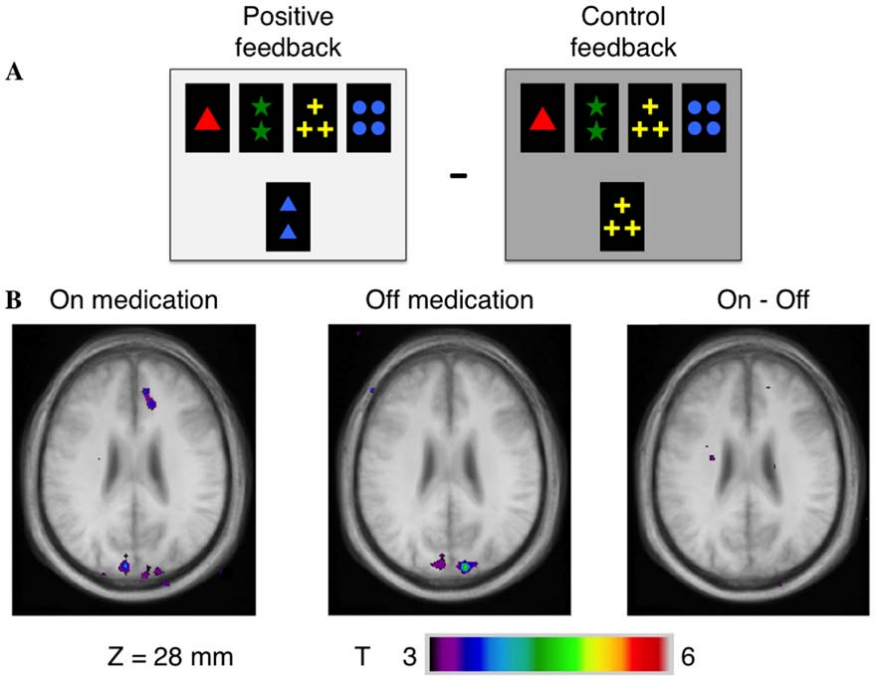
Patterns of activation in the right cingulate cortex when receiving positive feedback compared to control feedback. A – appearance of the monitor when receiving positive feedback and control feedback. B – axial section (z = 28 mm) in the ON and OFF groups and in the intergroup analysis showing greater activations in the ON – OFF comparison.

**ON state:** (Table 4, Figure 3) we found significant activity increase in the bilateral prestriate cortex (areas 17,18,19), in the left superior parietal lobule, precuneus (area 7) and DLPFC (area 9) and in the right rostral cingulated cortex (area 32). **OFF state:** Significant activation were virtually the same in the prestriate cortex and in the superior parietal lobule as in the ON state, but there were no increase of activity in the frontal regions. **Intergroup comparison:** we found no significant difference of activity

**Table 4:** Receiving positive feedback minus control feedback.

Anatomical area		ON medication			OFF medication			
		x, y, z	t-stat	Cluster	x, y, z		t-stat	Cluster
Anterior CC	(32) R	8, 40, 30	4.00	456				
DLPFC	(9) L	−44, 8, 38	3.93*	416				
SPL	(7) pcu	0, −82, 44	3.86	>10000				
	(7) R				12, −88, 48		4.55	>10000
Prestriate cortex	(17) L	−6, −92, 8	5.06	sc	−4, −86, 4		4.36	sc
	(17) R	14, −96, 2	3.99	sc	6, −86, 8		5.92	sc
	(18) L	6, −82, 4	6.55	sc	6, −68, 0		5.12	sc
	(18) R	−6, −76, 8	5.66	sc	4, −74, 0		4.22	sc
	(19) L	−6, −90, 30	4.08	sc	−16, −56, 0		3.80	sc
	(19) R				12, −90, 28		4.81	sc
		ON greater than OFF			Off greater than ON			
		-	-	-	-	-		-

Abbreviations, same as Table 2.

### 4) Matching after positive feedback vs. control matching

**Figure 4:**
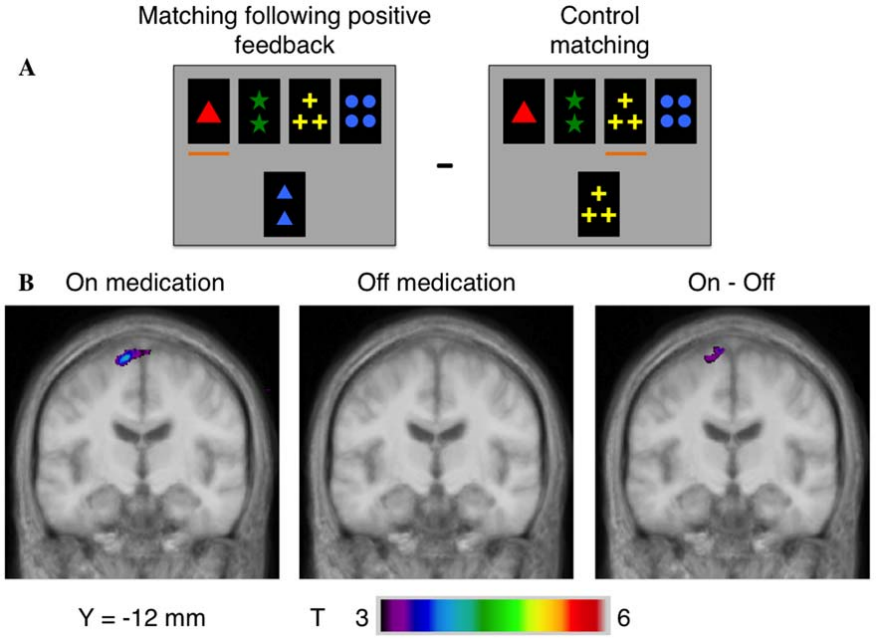
Patterns of activation in the left PMC when matching after positive feedback compared to matching in the control condition. A – appearance of the monitor when matching after positive feedback and matching in the control condition. B – coronal section (y = −12 mm) in the ON and OFF groups and in the intergroup analysis showing greater activations in the ON – OFF comparison.

**ON state:** (Table 5, Figure 4), we found significant activity increases in the left PMC (area 6) and in the medial PPC (area 7). **OFF state:** increases of activity were found in the left PPC (area 7) and in the bilateral prestriate cortex (area 18,19). **Intergroup comparison:** we found only a marginal increase of activity in the left prestriate cortex when comparing the OFF to the ON state.

**Table 5:** Matching after positive feedback minus control matching.

Anatomical area		ON medication			OFF medication		
		x, y, z	t-stat	Cluster	x, y, z	t-stat	Cluster
PMC	(6) L	−24, 10, 68	4.99	2480			
		−10, −12, 74	4.14	sc			
PPC	(7)	0, −54, 64	4.00*	368			
	(7) L				−26, −66, 58	5.13	840
Prestriate cortex	(18/19) L				−18, −92, 16	4.25	576
	(18/19) R				30, −84, 26	4.14	496
		ON greater than OFF			OFF greater than ON		
PMC	(6) L	−8, −12, 78	3.61*	104			
Prestriate cortex	(18/19) L				−44, −86, 2	3.87*	440

Abbreviations, same as Table 2.

### 5) Matching after negative feedback vs. matching after positive feedback

We computed that additional contrast to specifically assess the effect of L-Dopa on the left PMC, which was found in ON contrasts (2) and (4) (Table 6). Significant activation was found in the left premotor area in the ON but not in the OFF state. The intergroup comparison did not show increase of activity in the left premotor cortex.

**Table 6:** Matching after negative feedback minus matching after positive feedback (partial).

Anatomical area		ON medication			OFF medication		
		*x, y, z*	*t*-stat	Cluster	*x, y, z*	*t*-stat	Cluster
PMC	(6) L	−26, 12, 66	4.26	888	-	-	-
		ON greater than OFF			OFF greater than ON		
		-	-	-	-	-	-

Abbreviations, same as Table 2. We only report here the left premotor cluster of activation.

## Discussion

The goal of the present study was to assess the extent to which L-Dopa medication restores normal patterns of activation in the cognitive and motor cortico-striatal loops in the context of WCST set-shifting. We expected L-Dopa to help restore the patterns of activation observed in controls [10], only when the putamen was required, i.e. during set-shifts.

**Figure 5:**
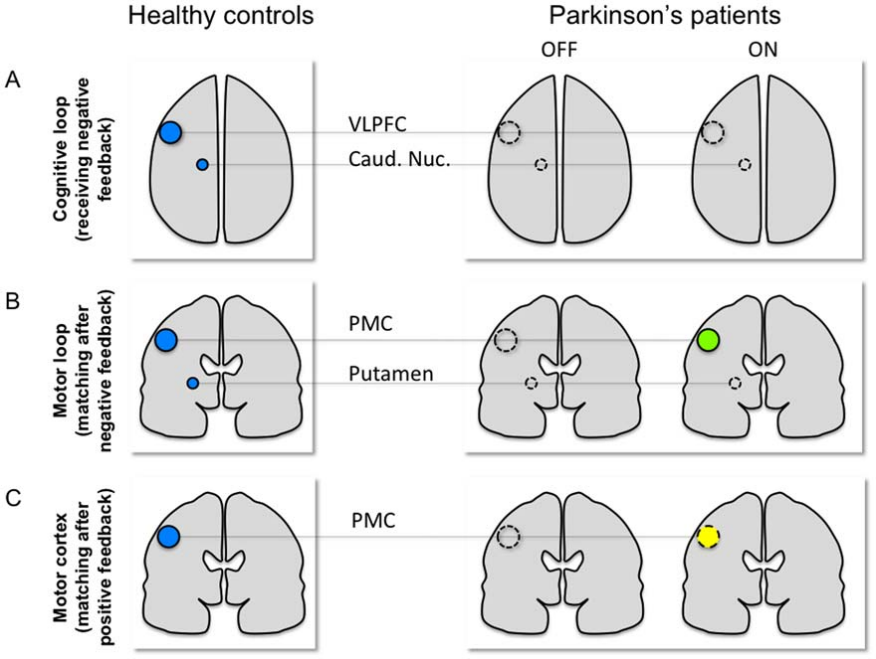
Explanatory diagram of the major results of the present study. A, In healthy controls (left) a cognitive cortico-striatal loop including the VLPFC and the caudate nucleus is significanty solicited when receiving negative feedback [10]. This activity is significantly reduced in PD patients OFF medication [8] and L-Dopa does not help restore the pattern of activation observed in control. B. In healthy controls (left), a motor cortico-striatal loop including the PMC and the putamen is significantly solicited when matching after negative feedback [10]. This activity is significantly reduced in PD patients OFF medication [8], and L-Dopa significantly restores the PMC activity (green circle) but not the putamen one. C. In healthy controls (left), the PMC is significantly activated without the putamen when matching after positive feedback [10]. This activity is significantly reduced in PD patients OFF medication [8], and L-Dopa partially restores the PMC activity (yellow dashed circle).

The key findings of this study in relation to our hypothesis is that, as predicted, L-Dopa medication restored the function of the motor cortico-striatal loop, during the execution of a set-shift since significantly more activation was found in the PMC during the ON vs. the OFF state when matching after negative feedback was compared with control matching (Figure 5). While an increase in activity was also observed in the PMC in the ON vs. the OFF state, when matching following positive feedback vs. control matching, the effect was not as large. Indeed, it was also found significantly more activated in the ON state only when comparing directly “matching after negative feedback” with “matching after positive feedback”. The “matching after negative feedback” condition was shown to rely on the motor cortico-striatal loop and, unlike “matching after positive feedback”, required putaminal activation in young healthy adults [10] (Figure 5). The computation of an additional contrast, namely “matching after negative feedback vs. matching after positive feedback”, revealed an activation of the left PMC in the ON and not in the OFF state establishes that L-Dopa medication had a stronger effect when the putamen was required by the task than when it was not.

By contrast, the medication did not restore the cognitive loop activity observed in controls when planning the set-shift. The “receiving negative feedback” condition was shown to rely on the cognitive loop [10] (including caudate nucleus and VLPFC, Figure 5). This result could be explained by the fact that the participants were at an early stage of the disease and exhibited signs of mild cognitive impairments at the maximum, but no signs of dementia, as assessed by the MoCA test. This suggests that the level of dopamine in the caudate nucleus may not have reached a functionally disabling threshold in the patients studied here, which explains the similar behavioral results between the ON and OFF states. Furthermore, the current results showed that L-Dopa medication had no direct significant effect on striatal activity, since no significant activation was found in this region for any of the experimental contrast in either of the two states (ON and OFF).

These observations are in line with previous FDG-PET studies showing that unlike the PD-related motor pattern (PDRP), the PD-related cognitive pattern (PDCP) expression was not significantly altered by antiparkinsonian treatment with either intravenous L-Dopa or deep brain stimulation [21]. In these studies, network analysis in non-demented PD patients identified a spatial covariance pattern associated with cognitive function and significant correlations between this PDCP expression and performance on tests of memory and executive functioning. However, antiparkinsonian treatment failed to detect significant changes in PDCP expression despite concurrent improvement in motor ratings and reductions in abnormal PDRP activity. Additionally, levodopa treatment has been shown to induce a flow-metabolism dissociation (reduction of the cerebral metabolic rate for glucose and rise of cerebral blood flow) in the PDRP, especially in the putamen [22]. These findings might provide a physiological basis for the BOLD contrasts reported in the present study.

The lack of difference in performance on the WCST in this study may appear to contradict reports that have suggested that the cognitive profile of patients in the ON-state is improved compared with the OFF-state [28]. However, it has been reported that the effect of L-dopa on cognitive performance in PD patients can be both positive and negative depending on the patient or the task to be performed [29]. Most importantly, at least two prior studies have reported that L-Dopa does not change performance on the WCST in PD patients [28], [30].

We have previously observed cortical over-activity in PD vs. controls in conditions not significantly requiring striatum (i.e. matching after and receiving positive feedback) in controls [8], [9], and had proposed it may be due to a mesocortical dopamine deficiency. Other studies have reported that L-Dopa can help reduce this cortical over-activity at least in the DLPFC [26], [27]. However, such a pattern was not clearly observed in the present study. The effect of L-Dopa on the DLPFC activation varied with the specific task period that was performed, and overall L-Dopa had little effect on cortical regions that did not co-activate with the striatum for the task, which in turn implies that it did not significantly alter mesocortical dopamine function. Another interpretation of the cortical over-activity observed in PD compared with control subjects is that it reflects a possible compensatory effect [25], which would help explain why patients at the early stages of the disease such as those in the present study do not yet show significant cognitive impairments. This interpretation is supported by the presence of significant activation observed in the DLPFC in the patients OFF and not ON medication when matching after negative feedback. In the OFF state this DLPFC activation could reflect a compensation for the lack of required PMC involvement, while in the ON state this compensation is not required since the activation in PMC is restored.

Our findings may reflect the fact that at early stages of the disease, motor loop DA is depleted across all PD patients, as they have been diagnosed and medicated on the basis of their motor symptoms. The absence of enhanced cortical activity in the cognitive loop may reflect the fact that L-Dopa intake was tuned to the patients' motor symptoms and not cognitive symptoms. Rowe et al. [23] have proposed that L-Dopa medication can enhance or restore striatal functions while impairing frontal functions by overdosing mesocortical dopamine. One limitation of our study comes from the small population sample and the fact that some patients were prescribed different types of dopaminergic medication together with L-Dopa. Our results and conclusions could be extended with a larger sample, as we could correlate, in the motor and cognitive loops, the extent of activation gain provided by the L-Dopa medication to the onset of the disease.

In conclusion, our study shows the functional implications of the stronger dopamine depletion in the putamen than in the caudate nucleus that is thought to occur in the early stages of PD [24]. More specifically, L-Dopa has a significant effect on the cortico-putaminal (motor) loop and not on the cortico-caudatal (cognitive) loop. These results help explaining why L-Dopa therapy is more effective in controlling motor symptoms than cognitive deficits. In the future, this type of fMRI protocol will allow for studies focusing on the effect of medication directly oriented at cognitive deficits in PD, in order to develop different treatment strategies.
